# An Investigation on the Status of Implementation of Communications and Information Management System (MCI) in Khorasan Razavi Hospitals

**DOI:** 10.5539/gjhs.v8n5p110

**Published:** 2015-08-31

**Authors:** Saeed Shojaei, Fereshteh Farzianpour, Mohammad Arab, Abbas Rahimi Foroushani

**Affiliations:** 1 Department of Health Management and Economic, School of Public Health, International Campus, Tehran University of Medical Sciences, Tehran, Iran; 2 Department of Health Management and Economics, School of Public Health, Tehran University of Medical Sciences, Tehran, Iran; 3 Departments of Epidemiology and Biostatistics, Tehran University of Medical Sciences, Tehran, Iran

**Keywords:** Communications and Information Management System (MCI), Khorasan Razavi, hospitals

## Abstract

**Background and Objectives:**

The aim of this investigation is to determine the mean scores of the possibility of implementing the MCI standards in Khorasan Razavi hospitals, from the perspective of Managers, in order to provide a suitable model for evaluating and promoting the system.

**Methods:**

This was a Research and method (R&D) and Survey Research method, which is of the type of Cross- Sectional, descriptive-analytic Studies conducted in two steps in hospitals of Khorasan Razavi from July to December 2014. This study was approved by the Ethical Committee of Tehran University of Medical Sciences (TUMS) in 2013/6/10. About the nature and purpose of the study was explained to the participants. Were used to apply functional assessment, based on Accreditation Model. In order to collect data, two questionnaires were used, all of which were taken from the standards of MCI. The reliability and validity of the questionnaires were approved by experts. Cronbach’s alphas for the questionnaires were obtained to be (0.95, 0.86), respectively. In order to analyze information, statistical analyses, including one way ANOVA, and Independent sample t-test were used.

**Results:**

The mean scores of the possibility of implementing the MCI standards in Khorasan Razavi hospitals, were (51.6 and 12.27), respectively.

**Conclusions:**

According to half (43.8%) of managers, the MCI standards are applicable in hospitals of Khorasan Razavi; however, their application requires greater efforts by the hospitals.

## 1. Background

Providing care for patients, is a complex effort, which to a large extent, depends on the transfer of information. This transfer of information is done in relation to the community and their families, and other health care specialists (Ajami et al., 2006). Failing to inform, is one of the most common root causes of safety incidents (Rahnavard et al., 2003).

To provide coordinated and integrated services, health care organization cites information about the knowledge of each patient’s care, provided care, care outcomes, and their performance. With regard to cases, such as human resources, materials and financial resources, information is a resource that leaders should effectively and efficiently manage (Srafi Zadeh et al., 2005; Iran Nejad Parizi et al., 2007). Every organization seeks to acquire, manage and use information, to promote the patients’ efficiencies, as well as individual and overall performance of the organization. Over time, the organization becomes competent in the following areas.

1) Identifying information needs;
2) Information Management System Design;
3) Gathering and acquiring data and information;
4) Analyzing data and converting them into information;
5) Sending and reporting data and information;
6) Integrating and using information (Farzianpour et al., 2015).

Although computerization and other technologies have effectively and efficiently been promoted, the principles of the appropriate management of information, applies to all methods, whether via paper or based on electronics, these standards are designed in such a way that can be used identically for both non-computerized systems and future technologies (Stoner et al., 2003). Nowadays, information is considered to be one of the world’s most important sources of power. Information is the fundamental of decision-making and planning (Dehghan et al., 2004). Decision-making, in today’s turbulent environment, without continuous access to relevant information, in effect, creates confusion, “in particular, it is an action for responding to this need, in the information age.” It is obvious that this action would help managers, if it is efficiently and effectively planned and designed, and then it is established (Dehghan et al., 2004). The complexity of the environment inside and outside the organization has increased the need for information, and thus the information systems (Mohanty et al., 1999). The quality of managers’ decisions is directly related to the information available to them. If the role of information systems is considered to be the provision of the required information for users, especially managers, then it should be borne in mind that the information needs of managers are different at different levels. In other words, different levels of management should be considered in designing information systems, because it would affect both information resources and how to provide it (Dehghan et al., 2004; Shojae et al., 2015).

Implementation of Hospital information system (HIS) in hospitals, can be discussed and investigated from the two points of view; technological and Management information system (MIS) (Borzekowski, 2009).

First, HIS system is a type of technological change in providing services in hospitals. The second point of view indicates the role of Hospital management information system (HMIS) in terms of providing information to the management, in order for hospital activities to be more effective (Peter Walton, 2012). If accurate and comprehensive information is timely made available to managers, it will minimize the risk of making incorrect decisions. Therefore, the complex organizations in the current era should mobilize themselves, so that they can regularly collect, process and analyze a variety of information required for management, and quickly make them available to managers (Farzianpour et al., 2011). Nowadays, computers are used to provide such information in various fields. And management information systems have taken measures to provide information to the large extent, which are needed by management in different fields, and are more widely used especially in larger and more developed organizations (Iran Nejad Parizi et al., 2007). Managers at all organizational levels, have found out that computerized information systems can provide necessary information for effective operations. Nowadays, management information system (MIS) gains more and more importance in planning, decision making, and desirable control, day by day. The degree of success of the control system depends on how fast managers can obtain accurate information about what is done on schedule, and what has been deviated from its path (Yusof et al., 2006). The general goal of the management of communication and information technology (MCI) in the health sector, is to accelerate collecting, achieving and supporting the health system processes, and effective decision-making for managing this system; because preparing and providing health care services for society is very complex, and highly dependent on the information system. Another important point is that the health care services will gradually turn into information-based services and may be knowledge-based services (Hamborg et al., 2004). Therefore, it is necessary for electronic health systems to be seriously taken into consideration (Dehghan et al., 2004). The application of information technology in health care systems can help medical professions to increase the quality of health care services (Mohanty et al., 1999; Kimiafar et al., 2007). MCI international standards, which is a domain of JCI international standards, can help them find out how they can create real reform and improvement, to help their patients and reduce risks. The current study intends to analyze the status of MCI system in Khorasan Razavi hospitals, using the international standards of the management of communication and information (MCI). Using the results obtained from the research, an image of the current status of these hospitals can be provided, in this field, and an appropriate model can be designed to improve the quality of communication and information management system of the aforementioned hospitals. This model can help the managers and officials of the Ministry of Health and Medical Education with future planning.

## 2. Methods

This was a Research and method (R&D) and Survey Research method, which is of the type of Cross- Sectional, descriptive-analytic Studies conducted in two steps in hospitals of Khorasan Razavi from July to December 2014. This study was approved by the Ethical Committee of Tehran University of Medical Sciences (TUMS) in 2013/6/10. About the nature and purpose of the study was explained to the participants. The subjects signed the informed consent form to participle in the study. In the first step, the applicability of the standards in the study environment was assessed with a questionnaire comprised of the MCI standards and 28 questions with three choices (applicable, relatively applicable, and inapplicable). The questionnaire contained 6 questions in the domain of standards for the field of communication with society, 2 in the domain of standards for the field of communication with patient and their families, 21 in the domain of standards for the field of notification among the suppliers inside and outside the organization 6 in the domain of standards for the field of notification among the suppliers inside and outside the organization, and 3 in the domain of standards for the field of leadership and planning,14 in the domain of standards for the field of total data and information. The questionnaires were completed by overall Cranach’s “was determined to be 0.95 for the first questionnaire. Then, in order to determine the impact of each question, the coefficient was calculated with omission of one question at a time. The findings indicated that the coefficients varied from 0.85 to 0.86 and omission of each question alter the coefficient significantly. Thus, the applicability of all standards was established and the second questionnaire used all measurement elements. In the second step, the sample size was determined in such a fashion as to allow a maximum error of estimation of 1 with a confidence of 95%. Given the fact that there are 16 hospitals supervised by the hospitals of Khorasan Razavi, the sample size was determined to be 64 so that 4 individuals in each hospital, i.e. senior managers (manager and nursing manager) and personnel of office of clinical governance, completed the questionnaires. In two hospitals, however, due to presence of only one person in the office of clinical governance, only three questionnaires were completed, yielding a total of 62 questionnaires completed. The second questionnaire consisted of measurable elements of MCI standards in the form of 63 questions with Yes/No answers. The questions consisted of 19 questions in the domain of standards for the field of communication with society, 5 in the domain of standards for the field of communication with patient and their families, 22 in the domain of data standards for the field of notification among the suppliers inside and outside the organization, and 7 in the domain of standards for the field of leadership and planning, 24 in the domain of data standards for the field of the patients’ clinical records 14 in the domain of standards for the field of total data and information. In order to determine the content validity of the questionnaires, opinions and suggestions of professors and experts of management of healthcare services were used. Regarding the reliability of questionnaires, the SPSS software version 11 determined the value of Cronbach’s to be 0.95 for the first questionnaire and 0.86 for the second questionnaire. Data analysis was accomplished using SPSS software version 11.5 and statistical tests of one way ANOVA and t-test. The level of significance was fixed at 0.5.

### 2.1 Ethical Considerations

All participants were given a full explanation of the study and freely consented to participate in the research. The questionnaires did not contain the names of the participants and they were assured that the information collected would be kept confidential and under no circumstances would the published results contain the names of the participants.

## 3. Results

The 16 hospitals studied consisted of 4 (25.8%) general hospitals and 12 (74.2%) were specialized hospitals. The number of beds in hospitals ranged from 69 to 537, with a mean value of 249.7 and standard deviation of 145.6. In the present study, 25 men (40.32%) and 37 women (59.67%) completed the questionnaires. The field of study was management for 13 (20.9%), medicine and nursing for 31 (50%) and others for 18 (29.1%) of respondents. 16 (25.8%) questionnaires were completed by hospitals managers, 16 (25.8%) were completed by hospital matrons and 30 (48.4%) were completed by personnel of the office of clinical governance. The findings of the study, separated for each domain are as follows:

**Domain of standards for the field of communication with society:** Then mean and standard deviation of score for this domain were 16.46 and 3.41, respectively. In this domain, the measurable elements “Is the standards for the field of communication with society program being implemented in the hospitals?” and “Does the standards for the field of communication with society program influence the designing of hospitals procedures?” received positive answers from 61 (98.4%) respondents. The least rate of positive answer pertained to the measurable element “Is notification achieved through efficient media on a conventional and legal basis?” with 44 (71%) positive responses.

**Domain of standards for the field of communication with patient and their families:** The mean and standard deviation of score in this domain were 4 and 1.48, respectively. 55 (88.7%) of respondents answered positive to the measurable element “Do managers implement therapeutic protocols for conduct of procedures of communication with patient and their families?” while 46 (74.2%) answered positive to the measurable element “Are tools and principles of quality improvement used for designing new procedures or modifying current procedures?”

**Domain of standards for the field of notification among the suppliers inside and outside the organization:** In this domain, the mean and standard deviation of score were 7.6 and 2.76, respectively. The measurable elements “Do managers consider among the suppliers inside and outside the organization?”, “Are the results of monitoring submitted to supervisors, as well as managerial and supervisory authorities in a periodic fashion?” and “Are the data resulting from clinical monitoring used for evaluation of the improvement process?” received positive answers by 52 (83.9%) respondents while 36 (58.1%) responded positively to the measurable element “Is there a score identified for each scale?”

**Domain of** **standards for the field of leadership and planning:** In this domain, the mean and standard deviation of score were 16.9 and 5.20, respectively. 59 (95.2%) respondents answered positive to the measurable element “Are MCI analyzed?” whereas 40 (64.5%) gave positive answer to the measurable element “Are leadership and planning program MCI analyze?”

**Domain of standards for the field of the patients’ clinical records:** In the improvement domain, the mean and standard deviation were 6 and 1.49, respectively. The measurable element “Are domains prioritized by hospital managers considered in the reformative activities?” received 58 (93.5%) positive responses. 41 (66.1%) respondents answered positive to the measureable element “Are changes tried before implementation?”

**Domain of standards for the field of total data and information:** In general, the mean and standard deviation of MCI scores in the hospitals studied were 51.6 and 12.27, respectively. The mean scores of general hospitals were lower compared to specialized hospitals in domains of leadership and planning, analysis of monitoring data, improvement and MCI. However, the general hospitals scored higher in domains of designing clinical and managerial procedures and data collection for monitoring quality (Table 1).

**Table 1:** Mean and standard deviation of scores of each domain and MCI for type of hospital in hospitals of in Khorasan Razavi

Domain	Type of Hospitals	X±SD
The standards for the field of communication with society	General	15.9± 2.7
	Specialized	16.8 ±3.61

The standards for the field of communication with patient and their families	General	4.06± 1.3
	Specialized	3.93± 1.5

The standards for the field of notification among the suppliers inside and outside the organization	General	7.8 ±1.9
	Specialized	7.5± 3

The standards for the field of leadership and planning	General	16.4± 3.9
	Specialized	17± 5.6

The standards for the field of the patients’ clinical records	General	5.8 ±1.5
	Specialized	6 ±1.4

The standards for the field of total data and information	General	50.2± 8.2
	Specialized	51.3± 13.4

**Evaluation of Factors Affecting Scores of Domains and MCI:**

According to the t-test, the type of hospital and gender of respondents do not affect any of the domains and MCI (Tables 2, 3). Analysis of variance indicated that the position of respondents does not influence the domains and MCI (Table 4).

**Table 2:** Mean and standard deviation and results of t-test related to scores of each domain and MCI for type of hospital in hospitals of Khorasan Razavi in 2015

Domain	N	Type of Hospitals	X±SD	P-value
The standards for the field of communication with society	46	General	16.89±3.61	0.34
	16	Specialized	15.93±2.74	

The standards for the field of communication with patient and their families	46	General	3.97±1.54	0.84
	16	Specialized	4.06±1.34	

The standards for the field of leadership and planning	46	General	7.56±3.00	0.70
	16	Specialized	7.87±1.96	

The standards for the field of notification among the suppliers inside and outside the organization	46	General	17.06±5.60	0.68
	16	Specialized	16.43±3.96	

The standards for the field of the patients’ clinical records	46	General	6.06±1.48	0.56
	16	Specialized	5.81±1.55	

The standards for the field of total data and information	46	General	51.34±13.44	0.76
	16	Specialized	50.25±8.28	

**Table 3:** Mean and standard deviation and results of t-test related to scores of each domain and MCI for gender in hospitals of Khorasan Razavi in 2015

Domain	Gender	N	X±SD	P-value
The standards for the field of communication with society	Male	25	16.12±4.55	0.38
	Female	37	17±2.36	

The standards for the field of communication with patient and their families	Male	25	4.04±1.30	0.86
	Female	37	3.97±1.60	

The standards for the field of leadership and planning	Male	25	7.40±2.62	0.57
	Female	37	7.81±2.87	

The standards for the field of notification among the suppliers inside and outside the organization	Male	25	17.16±5.08	0.75
	Female	37	16.72±5.35	

The standards for the field of the patients’ clinical records	Male	25	6.16±1.01	0.36
	Female	37	50.48±13.62	

The standards for the field of total data and information	Male	25	51.45±11.44	0.76
	Female	37	50.25±8.28	

**Table 4:** Mean and standard deviation and results of t-test related to scores of each domain and MCI for organizational position of respondents in hospitals of Khorasan Razavi in 2015

Domain	Indices & Test Results Domain				

	Manager	Matron	Clinical Governance	Total	Test Result
	X±SD	X±SD	X±SD	X±SD	P-value
The standards for the field of communication with society	15.75±4.75	17. 5±1.77	16.53±3.18	16.64±3.41	0.25
The standards for the field of communication with patient and their families	3.93±1.28	4.56±0.96	3.73±1.74	4±1.48	0.19
The standards for the field of leadership and planning	7.50±2.47	8.62±2.60	7.20±2.94	7.64±2.76	0.24
The standards for the field of notification among the suppliers inside and outside the organization	17±4.57	18.62±4.12	15.93±5.90	16.90±5.20	0.25
The standards for the field of the patients’ clinical records	5.93±1.61	6.12±1.45	5.96±1.49	6±1.49	0.92
The standards for the field of total data and information	50.12±12.96	55.68±9.31	49.1±12.98	51.06±12.27	0.21

Results of analysis of variance indicated that the number of beds affects the domains of standards for the field of communication with society (p=0.043), standards for the field of communication with patient and their families (p=0.007) and MCI (p=0.03).

Scheffe’s multiple comparison indicated that in the domain of standards for the field of notification among the suppliers inside and outside the organization, the mean scores of hospitals with fewer than 109 beds and those with more than 246 beds are significantly different (p=0.007), whereas the difference between mean.

MCI scores of hospitals with fewer than 109 beds and those with more than 246 beds is marginally significant (p=0.05). Analysis of variance indicated that the respondents’.

Analysis of variance indicated that the respondents’ field of study only affected the domain of the standards for the field of leadership and planning (p=0.004). Scheffe’s multiple comparison indicated that in the domain of data collection for monitoring quality, only a marginally significant difference is observed between the mean scores of those respondents who have studied medicine/nursing and those who have studied management.

## 4. Discussion

**Domain of standards for the field of communication with society:** Chaudhri et al states that the managers assume a particularly important role in any organization and knowledge of communication with society a major responsibility of managers alongside planning, organizing and controlling (Chaudhri et al., 2007). According to our findings, 53.3% of questionnaires received a score of 18-19; in other words, 33 (53.3%) respondents believed that the standards of this domain are being implemented excellently. Dhanesh (2012) reported the rate of observance of communication with society standards to be equal to 24% in the emergency wards of a general hospitals of Khorasan Razavi (Dhanesh, 2012). Azizi et al. (2010) and Moradi et al. (2009) reported the rates of observance of communication with society standards to be equal to 78%, 63% and 54% in three hospitals of Iran University of Medical Sciences (Azizi et al., 2010). Comparison with Iranian studies indicates that implementation of systems of quality management and models of excellence raise the score of communication with society.

**Domain of standards for the field of communication with patient and their families:** Patients’ health is affected by different healthcare procedures (Akbarian Bafghi, 2005; Hajavi et al., 2004). According to our findings, 61.3% of questionnaires scored 5, indicating that 38 (61.3%) respondents believe that the standards of this domain are being implemented excellently. Sanaz Amirifar et al. (2010) reported the rate of observance of clinical procedures standards to be equal to 27% in the emergency ward of a general hospital of Tehran University of Medical Sciences. The discrepancy between our study and the one mentioned above may be accounted for by the fact that the latter was conducted only in one ward, i.e. the emergency department, whereas our study evaluated the procedure designing in all hospitals (Sanaz Amirifar et al., 2010).

**Domain of standards for the field of notification among the suppliers inside and outside the organization:** Hospitals are required to present a report of their notification among the suppliers inside and outside the organization (Hajavi et al., 2004; Ebadi fardazar et al., 2006). Results of projects of notification among the suppliers inside and outside the organization are published extensively (Hajavi et al., 2004; www.jointcommissioninternational.org; Farzianpour et al., 2011, 2014, 2015), and these results influence the health policies significantly (Farzianpour et al., 2011). Presenting a report requires standards for the field of notification among the suppliers inside and outside the organization. Our findings indicate that 53.4% of questionnaires scored 9-10; in other words, 33 (53.4%) respondents believed that the standards of this domain are being implemented excellently. Sanaz Amirifar et al (2010) reported the rate of observance of standards of standards for the field of notification among the suppliers inside and outside the organization and standards for the field of leadership and planning to be equal to 39.5% and 29.3%, respectively, in the emergency ward of a general hospital of Tehran University of Medical Sciences (Sanaz Amirifar et al., 2010).

**Domain of standards for the field of leadership and planning:** Turani et al. (2010) reported the rates of observance of standards for the field of leadership and planning to be equal to 70%, 58.5% and 53% in three hospitals of Iran University of Medical Sciences (Turani et al., 2010).

The findings of the present study and other studies indicate that implementation of MCI and models of excellence and observing their requirements raise the score.

**Domain of standards for the field of the patients’ clinical records:** Nabilo and Donini have highlighted the role or participation of workers and increasing their creativity in organizations for the purpose of perpetual quality improvement for patients’ clinical records (Nabilo et al., 2005; Donini et al., 2008). According to our findings, 53.2% of questionnaires scored 7. In other words, 33 (53.2%) respondents believed that the standards of this domain are being implemented excellently. Sanaz Amirifar et al. (2010) reported the rate of observance of standards of improvement to be equal to 31.6% in the emergency ward of a general and special hospitals of Tehran University of Medical Sciences (Sanaz Amirifar et al., 2010). The findings of other similar studies do not corroborate those of our study, presumably due to the fact that those studies have dealt with one hospital only.

**Domain of standards for the field of total data and information:** Our study indicated that 45.1% of MCI questionnaires scored 56-63, indicating that 30 (45.1%) respondents believe that the standards of MCI are being implemented excellently. In a study by Turani et al. (2010) on hospitals of Iran University of Medical Sciences, the mean rates of observance of standards of quality improvement and patient safety were 72%, 57.6% and 57.4%. Among these, the hospital with an implemented EFQM excellence model had the highest score (Turani et al., 2010).

According to the findings of Raji Dargah et al. (2010), the patient safety of patients admitted in a specialized hospital of Tehran University of Medical Sciences was 68.16% (Raji Dargah et al., 2010). Amirifar et al. (2010) conducted a study in the emergency department of a general hospital of Tehran University of Medical Sciences to report that only 31.6% of standards of data and information in quality improvement and patient safety were observed completely, while 44.9% were observed relatively and 23.5% were not observed at all (Amirifar et al., 2010). Ammenwerth et al. evaluated Effect of a nursing information system on the quality of information processing in nursing in hospitals and concluded that expansion of capacity of quality improvement requires investments and education (Ammenwerth et al., 2011; Walton, 2012). Although the authors faced limitations for comparing the results with those of international studies, comparison with Iranian studies indicates that our hospitals are in an intermediate level regarding quality improvement and patient safety. Furthermore, hospitals that use excellence models and systems of quality management demonstrate a better status.

## 5. Conclusions

According to half (43.8%) of managers, the MCI standards are applicable in hospitals of Khorasan Razavi; however, their application requires greater efforts by the hospitals. Implementation and actualization of standards in hospitals require certain infrastructures such as better knowledge on the part of managers regarding the principles and tools of quality improvement, training personnel about the standards, implementation of models of quality management and organizational excellence, reinforcing the public affairs in hospitals and using hospital information system (MIC, HIS) all of which influence the process of realization for standards of quality improvement and patient safety.

**Limitations**

The limitations of this study included several changes in management of the State Welfare Organization of the province that delayed the implementation phase of the project. Other restrictions were the lack of cooperation by some managers for completing the questionnaire and it was necessary to fully explain all options to them.
